# Prevalence and genetic basis of *Mycobacterium tuberculosis* resistance to pretomanid in China

**DOI:** 10.1186/s12941-024-00697-0

**Published:** 2024-05-03

**Authors:** Bing Zhao, Huiwen Zheng, Juliano Timm, Zexuan Song, Shaojun Pei, Ruida Xing, Yajie Guo, Ling Ma, Feina Li, Qing Li, Yan Li, Lin Huang, Chong Teng, Ni Wang, Aastha Gupta, Sandeep Juneja, Fei Huang, Yanlin Zhao, Xichao Ou

**Affiliations:** 1 National Key Laboratory of Intelligent Tracking and Forecasting for Infectious Diseases, National Center for Tuberculosis Control and Prevention, Chinese Center for Disease Control and Prevention, Beijing, 102206 China; 2 Laboratory of Respiratory Diseases, Beijing Key Laboratory of Pediatric Respiratory Infection Diseases, Beijing Children’s Hospital, Key Laboratory of Major Diseases in Children, Ministry of Education, National Clinical Research Center for Respiratory Diseases, Beijing Pediatric Research Institute, Capital Medical University, National Center for Children’s Health, Beijing, 100045 China; 3 TB Alliance, New York, United States of America; 4 School of Public Health, Peking University, Beijing, 100191 China; 5 Institute of Tuberculosis Prevention and Control, Gansu Provincial Center for Disease Control and Prevention, Lanzhou, 730020 China; 6 Department of Tuberculosis Control, Chengde Center of Disease Prevention and Control, Chengde, 067000 China

**Keywords:** Pretomanid, Resistance, Breakpoint, Mutation

## Abstract

**Background:**

Pretomanid is a key component of new regimens for the treatment of drug-resistant tuberculosis (TB) which are being rolled out globally. However, there is limited information on the prevalence of pre-existing resistance to the drug.

**Methods:**

To investigate pretomanid resistance rates in China and its underlying genetic basis, as well as to generate additional minimum inhibitory concentration (MIC) data for epidemiological cutoff (ECOFF)/breakpoint setting, we performed MIC determinations in the Mycobacterial Growth Indicator Tube™ (MGIT) system, followed by WGS analysis, on 475 *Mycobacterium tuberculosis* (MTB) isolated from Chinese TB patients between 2013 and 2020.

**Results:**

We observed a pretomanid MIC distribution with a 99% ECOFF equal to 0.5 mg/L. Of the 15 isolates with MIC values > 0.5 mg/L, one (MIC = 1 mg/L) was identified as MTB lineage 1 (L1), a genotype previously reported to be intrinsically less susceptible to pretomanid, two were borderline resistant (MIC = 2–4 mg/L) and the remaining 12 isolates were highly resistant (MIC ≥ 16 mg/L) to the drug. Five resistant isolates did not harbor mutations in the known pretomanid resistant genes.

**Conclusions:**

Our results further support a breakpoint of 0.5 mg/L for a non-L1 MTB population, which is characteristic of China. Further, our data point to an unexpected high (14/475, 3%) pre-existing pretomanid resistance rate in the country, as well as to the existence of yet-to-be-discovered pretomanid resistance genes.

**Supplementary Information:**

The online version contains supplementary material available at 10.1186/s12941-024-00697-0.

## Background

Drug-resistant tuberculosis (DR-TB) remains a threat to public health. In 2021, approximately 450,000 new cases of rifampicin-resistant TB (RR-TB)/ multidrug-resistant TB (MDR-TB; defined as RR-TB with additional resistance to isoniazid) were reported worldwide [1]. Historically, RR/MDR-TB has been more difficult and costly to treat, necessitating more toxic and less effective drugs administered for a longer period of time, as compared to drug-susceptible TB (DS-TB) [2]. The recent introduction of novel drugs – the diarylquinoline bedaquiline (B) and the nitroimidazoles delamanid and pretomanid (Pa) – alongside the repurposing of the oxazolidinone linezolid (L), promise to revolutionize RR/MDR-TB therapy. In 2022, the World Health Organization (WHO) endorsed the BPaL and BPaLM (BPaL plus moxifloxacin) regimens for the treatment of RR/MDR-TB, based on data from clinical trials [3].

However, the worldwide rollout of BPaL/BPaLM regimens has been carried out despite limited information on the prevalence of resistance to these drugs, particularly the nitroimidazoles, which is mainly the result of not having rapid molecular tests. Pretomanid and delamanid are pro-drugs that share the same activation pathway, the products of *ddn, fgd1, fbiA-D* [4]. Loss-of-function and certain other mutations in any of these 6 genes have been associated with high delamanid/pretomanid resistance in *M. tuberculosis* (MTB) – in most cases, we see delamanid/pretomanid cross-resistance, but exceptions exist [5, 6]. In addition, other mechanisms must be involved in delamanid/pretomanid resistance, as strains exhibiting high minimum inhibitory concentration (MIC) to the drugs but no polymorphisms in these (canonical) genes have been identified [7, 8]. Because of their complex genetics, it will be difficult to develop rapid molecular tests for nitroimidazoles and, for the foreseeable future, we will most likely continue to rely on phenotypic drug susceptibility testing (pDST). pDST methods for both drugs have been described [9, 10], but a breakpoint (critical concentration) has been established only for delamanid [11, 12]. The definition of a breakpoint for pretomanid has been complicated by the discovery that one of the common MTB genotypes, lineage 1 (L1), is intrinsically less susceptible to the drug and shows a higher epidemiological cutoff (ECOFF), as compared to other lineages [10], but still lower than MICs of mutants in the canonical delamanid/pretomanid resistance genes. For the MTB L1 ECOFF to be assigned as the clinical breakpoint for all MTB lineages, it is necessary to consider treatment outcomes of patients harboring MTB L1 and treated with pretomanid-containing regimens. This type of data is still lacking.

In China, a country with high DR-TB burden, three previous studies have tested MTB clinical isolates against delamanid and/or pretomanid. Two of these studies employed the microplate Alamar Blue assay (MABA), which is not a WHO-recommended pDST method, did not present QC/reproducibility data or MTB lineage information [7, 13]. In the study by Zhang and collaborators, out of 72 MTB isolates tested, none exhibited high MICs for pretomanid; whereas in the study by Wen and coworkers, out of 220 MTB isolates analyzed, 7 (3%) and 3 (1%) were markedly resistance to delamanid and pretomanid, respectively. We used the well-established CRyPTIC UKMYC6 broth microdilution (BMD) plate for pDST [10, 12], followed by whole genome sequence (WGS) analysis [14], to test 1603 clinical MTB isolates selected from the Chinese 2015 National Drug Resistance Surveillance Collection. We reported resistance rates near 0.7%, 0.4% and 0.4% to delamanid, bedaquiline or linezolid, respectively [15]. Here, we performed pretomanid MIC determinations in the Becton and Dickinson Mycobacterial Growth Indicator Tube™ (MGIT) system [10], followed by WGS analysis, in 475 MTB isolated from Chinese TB patients between 2013 and 2020. The aims of this work were to further investigate the prevalence of pre-existing pretomanid resistance and its underlying genetic basis, as well as to generate additional MIC data for ECOFF/breakpoint setting in the MGIT system.

## Materials and methods

### MTB isolate Collection

**Fig. 1:**
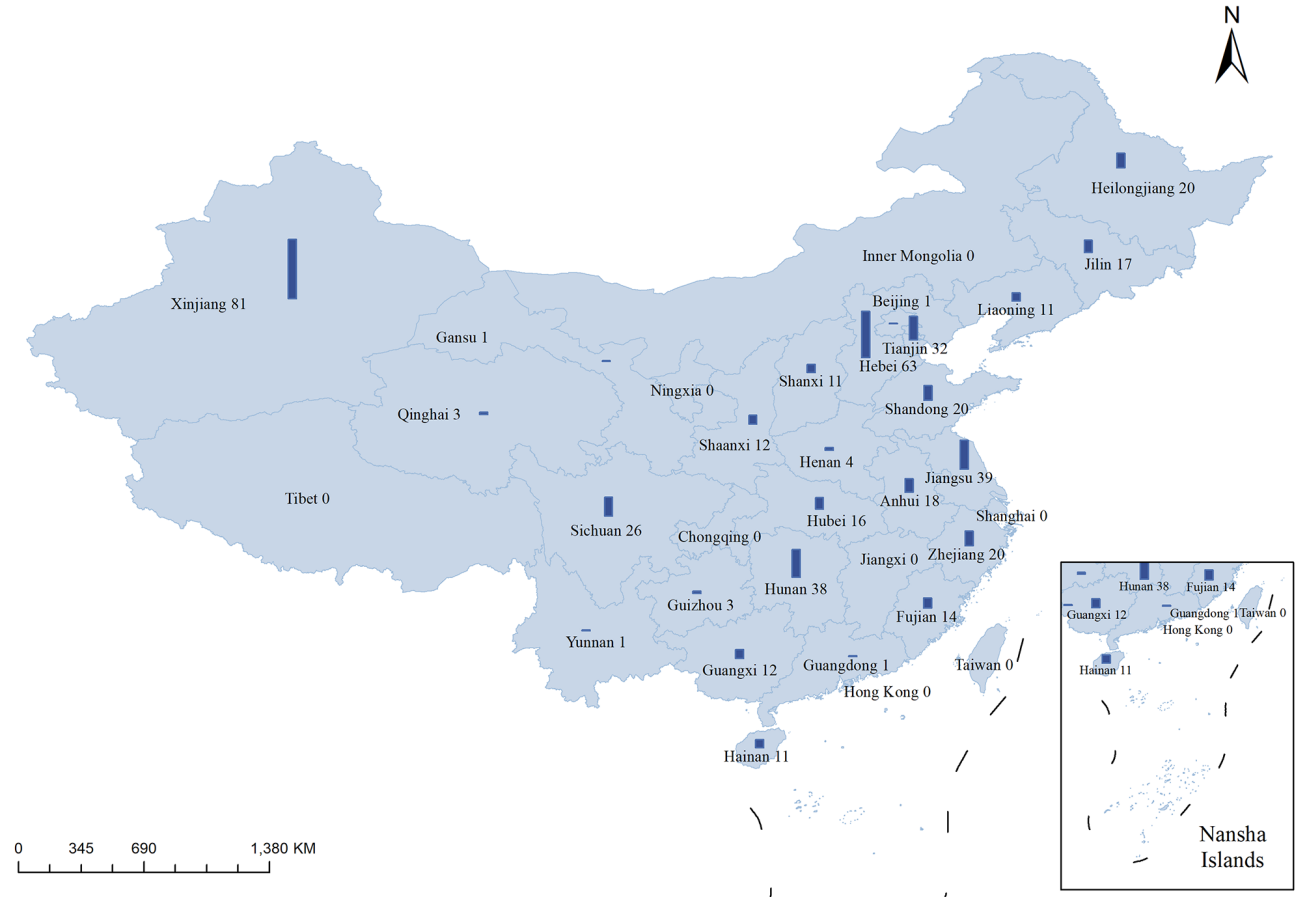
Geographical distribution of the 475 MTB isolates analyzed. Bars represent the number of MTB isolates originated from each province/municipality/autonomous region

MTB isolates were obtained from delamanid- and pretomanid-naïve TB patients from 21 provinces, 2 municipalities and 2 autonomous regions, corresponding to all 6 regions included in the Chinese Drug Resistance Surveillance Program running between 2013 and 2020 (Fig. 1; Supplementary Table). 15 isolates had been previously included in an analysis of the prevalence of delamanid resistance [15]. All isolates were stored frozen on 20% glycerol and sub-cultured on Löwenstein-Jensen medium prior to further analyses at the National Tuberculosis Reference Laboratory (NTRL).

### Broth microdilution DST

Phenotypic DST for a panel of 12 anti-TB drugs (amikacin, cycloserine, ethambutol, ethionamide, isoniazid [INH], kanamycin, moxifloxacin, ofloxacin, para-aminosalicylic acid, rifabutin, rifampicin [RIF] and streptomycin) was performed using the BMD method and a commercial dry plate, as previous described [10]. MIC was defined as the lowest concentration without obvious visible bacterial growth compared with positive controls.

MTB H37Rv (ATCC 27,294) strain was used to quality control (QC) all tests. The breakpoints for all 12 drugs were as described previously [12]. MDR isolates were defined as MTB resistant to at least INH and RIF, and pre-XDR were RR/MDR isolates with additional resistance to fluoroquinolones [16].

### Pretomanid MIC in the MGIT

The pretomanid MIC determinations were performed as described previously [10]. The concentrations used for MIC testing were 2-fold serial dilution from 0.03 mg/L to 2 mg/L. If the MIC fell outside of this range, the test was repeated at higher (2–16 mg/L) or lower (0.004–0.016 mg/L) concentrations to avoid truncating the phenotypically wild-type MIC distribution. H37Rv was included as reference in each batch of MIC testing. This generated 19 data points, which all fell within a 4-dilution range (0.06–0.5 mg/L; Supplemental Fig. 1). This QC range is identical to the one reported by Bateson and collaborators [10]. The ECOFF for pretomanid was determined using the European Committee for Antimicrobial Susceptibility Testing (EUCAST) software ECOFFinder (https://www.eucast.org/mic_and_zone_distributions_and_ecoffs).

### WGS Analysis

Genomic DNA was prepared using the cetyltrimethylammonium bromide method, then subjected to WGS using the Illumina HiSeq 2000 platform as described previously [17, 18]. All raw WGS data were processed with the Clockwork pipeline, originally developed for the CRyPTIC Consortium by the European Bioinformatics Institute [19]. Minimum coverage was 10 X. Sequences containing genes of the proline–glutamic acid (PE)/proline–proline–glutamic acid (PPE) family and other repetitive sequences were excluded from the analysis. Sequencing reads corresponding to *ddn*, *fbiA*-*D* and *fgd1* were aligned to those from the H37Rv reference genome (GenBank ID: NC_000962.3). Site statistics were generated using SAMtools mpileup and gene annotation generated using snpEff software. Phylogenetic tree was visualized and modified with iTOL (v 6.4.3).

## Results

### Characterization of the MTB isolate Collection

**Fig. 2:**
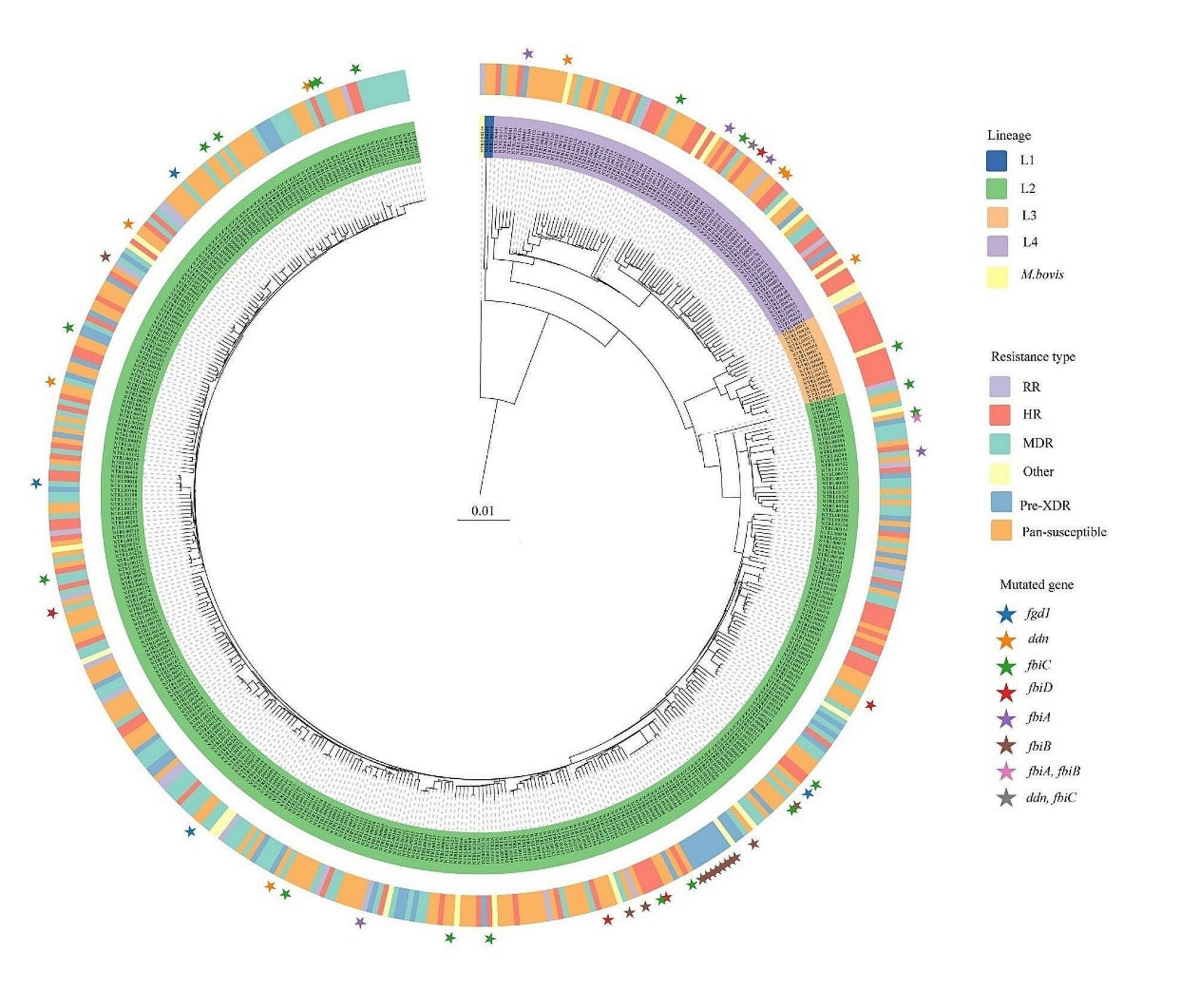
Phylogenetic tree. Maximum-likelihood tree for the 475 MTB isolates. Colors represent MTB lineage (inner circle), drug resistance type (outer circle). Non-synonymous mutations in canonical delamanid/pretomanid resistant genes are shown by asterisks

A total of 475 MTB isolates were randomly selected from the NTRL collection, including 171 (36%) isolates susceptible to all 12 drugs tested (pan-susceptible), 95 (20%) INH resistant (HR), 23 (5%) RR, 114 (24%) MDR and 48 (10%) pre-XDR isolates. The remaining 24 (5%) isolates were susceptible to INH and RIF, but resistant to at least one other drug included in the BMD plate (Supplementary Table). All the major MTB complex lineages were represented; from most abundant to least, lineage 2 (L2) (78.7%, 374/475), L4 (17.1%, 81/475), L3 (3.6%, 17/475) and L1 (0.4%, 2/475). In addition, one *Mycobacterium bovis* isolate was identified (Fig. 2, Supplementary Table).

### Pretomanid MIC distribution in the MTB isolates

**Fig. 3:**
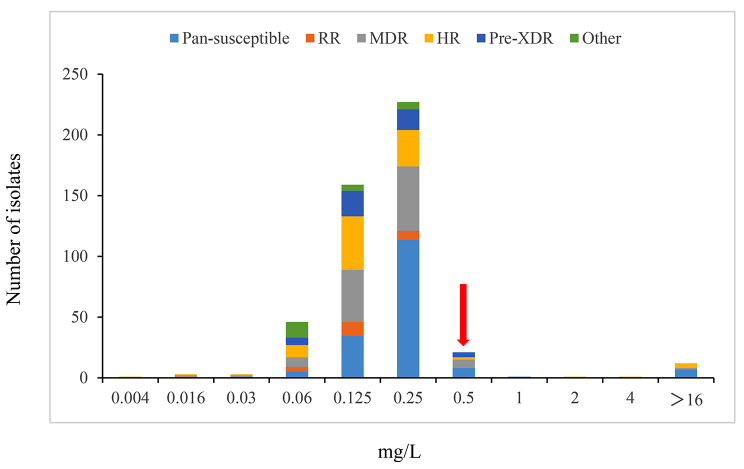
Distribution of Pretomanid MICs in MTB isolates. The arrow indicates the 99th percentile calculated using ECOFFinder. Colors indicate the different MTB resistance types

Testing of the 475 isolates for pretomanid revealed a MIC distribution with a mode equal to 0.25 mg/L and 99% statistical ECOFF equal to 0.5 mg/L (Fig. 3). Of the 15 isolates with MIC values > 0.5 mg/L, one had MIC = 1 mg/L and was identified as MTB L1 and 12 (2.5%) isolates had high MICs (≥ 16 mg/L) and can be considered resistant. The two (0.4%) remaining isolates, NTRL00030 and NTRL00212 had MICs equal to 2 mg/L and 4 mg/L, respectively, and were called “borderline” resistant. Both isolates belong to the L2 clade. There was no obvious correlation between pretomanid MIC levels and susceptibility to the 12 other anti-TB drugs tested (Table 1; Supplemental Table).

**Table 1:** Genetic analysis of the 14 pretomanid-resistant MTB isolates

Isolate ID	Resistance type	Sublineage	Gene [previous report reference]				Pa MIC (mg/L)	DLM resistance [15]
			ddn	fbiA	fbiC	fbiD		
NTRL00025	Pan-susceptible	L4.5	281_del_21_CAACCCC AAGGTTCAGGTACAG_C [NM]				> 16	R
NTRL00030	HR	L2.2.1					2	S
NTRL00032	Pan-susceptible	L2.2.1		T-85G [NM]			> 16	S
NTRL00034	HR	L4.5	281_del_21_CAACCCC AAGGTTCAGGTACAG_C [NM]				> 16	R
NTRL00200	HR	L2.2.1					> 16	ND
NTRL00212	HR	L2.2.1				Thr4Ala(aca/Gca) [NM]	4	ND
NTRL00216	HR	L2.2	Tyr29STOP(tac/taA) [NM]	A-153G [NM]			> 16	ND
NTRL00224	HR	L2.2.1		A-227G [NM]			> 16	ND
NTRL00320	Pan-susceptible	L4.4	Tyr89STOP(tac/taA) [21]				> 16	ND
NTRL00335	MDR	L4.5					> 16	R
NTRL00351	Pan-susceptible	L2.2.1.1	Leu49Pro(ctg/cCg) [4]				> 16	R
NTRL00391	Pan-susceptible	L2.2.1	Leu64Pro(ctc/cCc) [6]				> 16	R
NTRL00411	Pan-susceptible	L2.2.1	398_del_1_TC_T [NM]				> 16	R
NTRL00412	Pan-susceptible	L4.5	Ala77Thr(gcc/Acc) [22]		Gly839Ala(ggt/ggC) [22]		> 16	R

Abbreviations: del = deletion; HR = mono-INH resistant; MDR = multidrug-resistant; ND = not determined; NM = novel mutation; R = resistant; S = susceptible

Delamanid susceptibility data was available for 9 of the pretomanid-resistant isolates (Table 1). All, except for NTRL00030 and NTRL00032, had been shown to be also resistant to delamanid.

### Genomic analyses

Among the 460 pretomanid-susceptible (MIC ≤ 1 mg/L) isolates, we found 21 different synonymous and 35 nonsynonymous substitutions, affecting the 6 canonical pretomanid resistance genes, *ddn*, *fbiA-D* and *fgd1*. These polymorphisms were harbored by 412 (89.6%) and 48 (10.4%) isolates, respectively (Supplementary Table). Out of the 14 isolates borderline-resistant/resistant to pretomanid, only 9 isolates had non-synonymous or indel mutations in at least one of those genes, more specifically, *ddn, fbiA, fbiC and fbiD*. Three isolates showed single nucleotide polymorphisms (SNPs) upstream the coding region of *fbiA*, but they were positioned far from the putative start codon, thus unlikely to affect *fbiA* expression (Table 1).

Of note, NTRL00025 and NTRL00034 harbored the same 21 base-pair deletion in *ddn* and were both isolated from patients in Xinjiang Province. However, they were separated by 18 SNPs, including a fabG1 -15 C > T mutation conferring resistance to INH in NTRL00034; this genetic distance is greater than what has been seen in epidemiologically linked TB cases [20].

## Discussion

A widely accessible test for pretomanid resistance is urgently needed to determine pre-existing resistance rates in countries where the BPaL/BPaLM regimens are to be implemented, monitor resistance trends following implementation and, more importantly, for proper management of TB patients who are candidates to begin receiving these regimens, or who fail or relapse treatment with BPaL/BPaLM. Because of the complex genetics of pretomanid (and delamanid) resistance, rapid molecular tests will require considerable effort and time to develop. We are currently limited to pDST methods, which take weeks to produce a result and demand complex laboratory infrastructure but, at least some, are robust and accessible worldwide. pDST based on the MGIT system is currently the only commercial method that has been fully validated for pretomanid testing [10]; it is already in use for MIC testing in several countries [23]. However, a breakpoint (critical concentration) has yet to be recommended by WHO, although a screen value of ≤ 2 mg/L has been published by EUCAST [24].

Our study with 475 Chinese MTB clinical isolates provided additional pretomanid MGIT MIC data to be used for breakpoint setting. Basically, we confirmed the QC range (0.06–0.5 mg/L; Supplemental Fig.) for the test, as well as the ECOFF for MTB lineages other than L1 (0.5 mg/L; Fig. 3) first reported by Bateson and collaborators [10]. MTB L1 are not well represented in the Chinese population [25] and, indeed, only one isolate in our study proved to be MTB L1 (with an MIC = 1 mg/L; Supplemental Table). In addition to this isolate, 14 others had MIC > 0.5 mg/L (Table). These included NTRL00030 and NTRL00212, MTB L2 isolates with “borderline resistance” (MIC values of 2 and 4 mg/L, respectively). NTRL00030 had been found susceptible to delamanid previously and, here, was shown to carry wild-type canonical delamanid/pretomanid resistant genes (Table). We hypothesize that the slightly higher pretomanid MIC result in this isolate is simply the result of technical variation and may have no clinical impact. On the other hand, NTRL00212 was found to harbor a mutation in the *fbiD* gene, which may explain the borderline resistance phenotype; to our knowledge, this mutation has never been described (Table). All the remaining 12 isolates were resistant to the highest pretomanid concentration typically tested (16 mg/L). Interestingly, only 8 of these harbored a mutation in the canonical delamanid/pretomanid resistant genes. Also of note, NTRL00032 had been tested delamanid susceptible in our previous study [15]. These observations add to data formally published [7, 8], strongly suggesting the existence of other, still unidentified delamanid/pretomanid resistance genes and mutations leading to resistance to one but not the other nitroimidazole.

The prevalence of pretomanid resistance seen in this study, near 3%, is similar to the delamanid resistance rate we reported previously [15], but greater than rates reported elsewhere [26, 27]. This highlights the importance of ensuring that delamanid/pretomanid resistance is carefully monitored in China.

We also acknowledge some limitations in this study. First, the 475-isolate collection studied here does not provide a full picture of MTB circulating in China; some provinces/municipalities contributed few, if any, isolates; and there was only one MTB L1. Parenthetically, the determination of a critical concentration for pretomanid that includes MTB L1 will require clinical outcome data from cohorts of patients infected with this MTB genotype and treated with BPaL/BPaLM. Second, the novel mutations in pretomanid resistance genes identified here, except for the loss-of-function ddn_p.Tyr29STOP, must have their association to resistance confirmed by further experimental evidence, including larger WGS/pDST association studies, protein modeling and, ideally, allelic exchange experiments.

## Conclusions

Our study with 475 MTB isolates suggests that the rate of pre-existing resistance to pretomanid in China, 3%, is higher than expected, and that unknown genetic mechanisms may contribute to this. Follow-up studies with larger sample sizes are needed to further substantiate these findings.

### Electronic supplementary material

Below is the link to the electronic supplementary material.

Supplementary Material 1

## Data Availability

The datasets in the present study are accessible from the corresponding author, ZHAO YL.

## Abbreviations

ECOFF: Epidemiological cutoff
MIC: Minimum inhibitory concentration
MGIT: Mycobacterial Growth Indicator Tube™
DR-TB: Drug-resistant tuberculosis
RR-TB/MDR-TB: Rifampicin-resistant TB/ multidrug-resistant TB
DS-TB: Drug-susceptible TB
B: Bedaquiline
Pa: Pretomanid
L: Linezolid
WHO: World Health Organization
MTB: *Mycobacterium tuberculosis*
pDST: Phenotypic drug susceptibility testing
L: Lineage
MABA: Microplate Alamar Blue assay
BMD: Broth microdilution
WGS: Whole genome sequence
NTRL: National Tuberculosis Reference Laboratory
INH: Isoniazid
RIF: Rifampicin
QC: Quality control
EUCAST: European Committee for Antimicrobial Susceptibility Testing
PE/PPE: Proline–glutamic acid/proline–proline–glutamic acid
SNPs: Single nucleotide polymorphisms
